# Safe Decomposition of Startup Requirements: Verification and Synthesis

**DOI:** 10.1007/978-3-030-45190-5_9

**Published:** 2020-03-13

**Authors:** Alessandro Cimatti, Luca Geatti, Alberto Griggio, Greg Kimberly, Stefano Tonetta

**Affiliations:** 8 Johannes Kepler University, Linz, Austria; 9 University of Birmingham, Birmingham, UK; 10 Fondazione Bruno Kessler, Trento, Italy; 11 University of Udine, Udine, Italy; 12 The Boeing Company, Seattle, USA

## Abstract

The initialization of complex cyber-physical systems often requires the interaction of various components that must start up with strict timing requirements on the provision of signals (power, refrigeration, light, etc.). In order to safely allow an independent development of components, it is necessary to ensure a safe decomposition, i.e. the specification of local timing requirements that prevent later integration errors due to the dependencies. We propose a high-level formalism to model local timing requirements and dependencies. We consider the problem of checking the consistency (existence of an execution satisfying the requirements) and compatibility (absence of an execution that reaches an integration error) of the local requirements, and the problem of synthesizing a region of timing constraints that represents all possible correct refinements of the original specification. We show how the problems can be naturally translated into a model checking and synthesis problem for timed automata with shared variables. Exploiting the linear structure of the requirements, we propose an encoding of the problem into SMT. We evaluate the SMT-based approach using MathSAT and show how it scales better than the automata-based approach using Uppaal and nuXmv.

### 1 Introduction

Complex industrial cyber-physical systems often have an initialization procedure that requires to reach a startup mode within a specified design target time interval. In order for the system as a whole to complete the startup within the required interval, each subcomponent of the system may have to go through a number of intermediate phases, within their own target intervals, each of which may itself be dependent upon other subcomponents reaching startup or intermediate phases. E.g. for a power generation system to startup at full power, it may need to transition first through a low power output phase and a number of subsidiary systems (perhaps cooling or fuel supply) may first have to undergo their

A. Biere and D. Parker (Eds.): TACAS 2020, LNCS 12078, pp. 155–172, 2020.
https://doi.org/10.1007/978-3-030-45190-5_9

own phase transitions. In turn, these subsidiary systems may require transitions to occur in systems subsidiary to them and so on.

Traditionally, the integration of these distributed transition targets are validated via simulation and testing, which while sufficient to reach a desired design performance are labor and time intensive. Having a more efficient process for arriving at and validating a set of design targets that satisfy the overall system requirements is clearly beneficial in these contexts. Firstly, we would like to verify that these requirements prevent failed transitions in which the time performance of the subsidiary systems lead to outcomes where our main system (e.g., the power generation system) cannot perform a transition within its time window. For example, suppose the power system has a time window within which it must transition from low-power mode to high-power mode; in order for it to achieve this transition, however, it requires that two subsidiary systems, a cooling system and a fuel supply system, must themselves transition from a low-output mode to a high-output mode, each within their own target transition time windows. If these time windows are not compatible, the power generator may fail to provide the high power in time. Secondly, if our starting set of requirements is inadequate to provide this guarantee, we would like to be able to synthesize a set of requirements that is adequate to this task.

In this paper, we formalize the problem starting from a simple industrially relevant setting, where the components have a linear sequence of phases, must progress to the next phase within a certain interval of time, and must respect some dependencies upon the phases of other components. Dependencies are expressed as Boolean combinations of variables representing the component phases and are divided into two types: (i) *signal dependencies*, where the entering of a component into a phase is conditioned by the presence of other components in some specific phases; (ii) *state dependencies*, where a component can stay in a phase only if, during all its stay, other components are in some specific phases. We are interested in the following problems: 1) checking if the requirements are compatible, *i.e.*, if all reachable states can be extended with an execution satisfying the requirements; thus, if the components satisfy the local requirements, they cannot lead the system to an illegal state (where a component does not receive the input in time); 2) checking if the requirements are consistent, *i.e.*, there exists an execution of the components satisfying all requirements (inconsistency is actually a pathological case of incompatibility); 3) synthesizing the set of refinements (same requirements with stricter intervals) that are consistent and compatible. We show how the first two verification problems can be naturally translated into a model checking problem for timed automata with shared variables. Exploiting the linear structure of the requirements, we propose an encoding of the problem into SMT. If all intervals are bounded, the encoding is quantifier-free. Finally, both approaches have been extended to solve also the synthesis problem, using synthesis for parametrized model checking of TAs and quantifier elimination in SMT, respectively.

We implemented the SMT-based approach in a tool called TRICker and carried out experimental evaluation, comparing it with other tools for the verification of timed automata. We used Uppaal [6] and nuXmv [7] to model check TAs and MathSAT [12] to solve the SMT problems. We performed an experimental evaluation based on a test-set of randomly generated local requirements. When comparing the SMT-based approach with the automata-based one, the results highlight a better performance of the former technique on all three problems.

*Related Work* The problem of the integration and compatibility of input/output timed automata has been extensively studied in the literature. Typically, works in the literature focus on deadlock checking (see, e.g., [4,5]). The work of [2] also addresses the parameter synthesis to avoid deadlocks in timed automata. In order to check for livelocks, liveness properties can be addressed with approaches proposed in [10,7]. A general definition of illegal states for timed interface automata is given in [13]. As shown in the extended version of the paper the compatibility problem addressed in this paper can be seen as a subcase of the homonym problem for input/output timed interface automata. As we are considering a closed system, the problem reduces to the existence of a deadlock or livelock in a phase of some component (depending if the related time interval is bounded or not). Moreover, compared to the above model checking approaches we are considering a specific fragment of timed automata with a linear structure that can be exploited for specialized solutions.

Related problems have been addressed in the context of task scheduling. In the formalism introduced in [16,17], called DRT (short for *digraph real-time task* model), in which tasks and deadlines are expressed as directed graphs, the problem of determining whether a schedule exists (*feasibility problem*) bears some similarities with the consistency checking problem we study here. The DRT model allows the use of very general graph topologies, with multiple outgoing branches and loop-backs, but it does not consider dependencies across different tasks. The main difference with our work is that the problem is addressed from a *global* point of view (*i.e.*, the existence of a global scheduler that can coordinate the execution of the tasks), whereas we are interested in local solutions, in which each requirement can be considered in isolation. Another difference is the approach used to tackle the problem: while in [16] dynamic programming is used to deal with the possible explosion of the search space, we use SMT [14] as the main framework for all the three above-mentioned problems.

*Outline.* In Sec. 2, we introduce a suitable formalism to model local requirements and we formalize the three problems. In Sec. 3, we propose the reductions of *compatibility checking* and *consistency checking* into TAs and SMT. The corresponding solutions for the *synthesis* problem are then described in Sec. 4. The experimental results are described in Sec. 5. In Sec. 6, we draw some conclusions and highlight possible future directions of this work.

### 2 Problem Statement

*Domain formalization* We propose a high level formalism to model the local requirements.

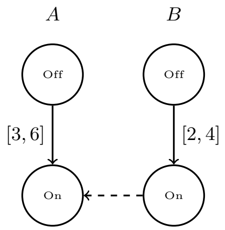

(a) Example of system with two local requirements and one state dependency.

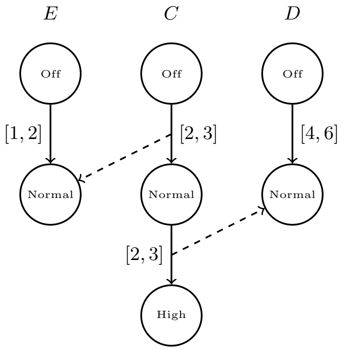

(b) Example of system with two local requirements and two signal dependencies.

**Definition 1 (Local Requirements)** *A specification $S$ is given by a set of local (or component) requirements, where each local requirement $C \in S$ is given by an (ordered) sequence $\langle P_1^C, \ldots, P_n^C \rangle$ of phases. In turn, each phase $P_i$ of $C$ is associated when $i > 1$ with a closed real interval $\beta_{P_i}$ with non-negative lower limit $l_{P_i}$ and (finite or infinite) upper limit $u_{P_i}$, with a formula $\phi_{P_i}$ (called* signal dependency*) and, when $i > 0$ with a formula $\psi_{P_i}$ (called* state dependency*). Both $\phi_{P_i}$ and $\psi_{P_i}$ are Boolean formulae over the atoms in $\{\langle D, Q\rangle\}_{D \in S \setminus \{C\}, Q \in D}$ (i.e., the phases of other components).*

If a dependency $\psi_P$ is just a conjunction of atoms, then we say that $\psi_P$ is *convex*. With the notation $|C|$, we will refer to the number of phases of $C$.

Figs. 1a and 1b show two examples of sets of local requirements. In Fig. 1a, we have two local requirements $A$ and $B$ (i.e., $S = \{A, B\}$); each local requirement has two phases *Off* and *On* (i.e., $P_1^A = Off$ and $P_2^A = On$ and similarly for $B$); the bounds are depicted in square brackets (thus, for example $\beta_{On}^A = [3, 6]$); all dependencies are trivially true apart from the state dependency $\psi_{On}^B = \langle A, On\rangle$ of the local requirement $B$, which is plotted as an arrow from the phase *On* of $B$ to phase *On* of $A$. In Fig. 1b, we have another example with three components and some signal dependencies; for example, signal dependency $\phi_{Normal}^C = \langle E, Normal\rangle$ is plotted as an arrow from the *transition* to phase *Normal* of $C$ to phase *Normal* of $E$.

**Definition 2 (Stronger local requirements)** *We say that a local requirement $C' = \langle P_1^{C'}, \ldots, P_n^{C'}\rangle$ is* stronger *than $C = \langle P_1^C, \ldots, P_n^C\rangle$ (written $C' \preceq C$), iff phase $P_i^{C'}$ is identical to $P_i^C$ except that $l_{P_i^C} \leq l_{P_i^{C'}}$ and $u_{P_i^{C'}} \leq u_{P_i^C}$, for all $1 \leq i \leq n$. Given two specifications $S = \{C_1, \ldots, C_n\}$ and $S' = \{C_1', \ldots, C_n'\}$, we say that $S'$ is stronger than $S$ (written $S' \preceq S$) iff for all $i$, $1 \leq i \leq n$, $|C_i| = |C_i'|$ and $C_i' \preceq C_i$.*

In defining the semantics of a composition of local requirements $C_1 \ldots C_n$, every local requirement $C_i$ is associated with a local clock, which is reset each time it enters a new phase. Given a local requirements specification $\{C_1, \ldots, C_n\}$, we define its semantics formally by defining the predicate $Reach((C_1, j_1, t_1), \ldots, (C_n, j_n, t_n))$, which is true iff the phases $P^{C_1}_{j_1} \ldots P^{C_n}_{j_n}$ are reachable at local times $t_1 \ldots t_n$.

**Definition 3 (Reachability for local requirements)** *Given the specification* $\{C_1 \ldots C_n\}$ *and the time points* $t_1 \in \mathbb{R} \ldots t_n \in \mathbb{R}$*, we inductively define the predicate* $Reach((C_1, j_1, t_1), \ldots, (C_n, j_n, t_n))$ *as follows:*

- (base case) $Reach((C_1, 1, 0), \ldots, (C_n, 1, 0))$ *holds and for all* $i \in \{1 \ldots n\}$ *it holds that (state dependencies):* $((C_1, 1), \ldots, (C_n, 1)) \models \psi^{C_i}_1$
- (timed transition) *if* $Reach((C_1, j_1, t_1), \ldots, (C_n, j_n, t_n))$ *and there exists a* $\delta \in \mathbb{R}$ *such that* $t_i + \delta \leq u^{C_i}_{j_i+1}$ *for all* $i \in \{1 \ldots n\}$*, then* $Reach((C_1, j_1, t_1 + \delta), \ldots, (C_n, j_n, t_n + \delta))$.
- (discrete transition) *if* $Reach((C_1, j_1, t_1), \ldots, (C_n, j_n, t_n))$ *and there exists a* $\delta \in \mathbb{R}$ *and a* $M \subseteq \{1, \ldots, n\}$ *such that:*
  1. *for all* $i \in \{1 \ldots n\}$ *such that* $j_i < |C_i|$, $t_i + \delta \in [l^{C_i}_{j_i+1}, u^{C_i}_{j_i+1}]$ *if* $i \in M$*, and* $t_i + \delta \leq u^{C_i}_{j_i+1}$ *otherwise;*
  2. *for all* $i \in M$*, it holds that (signal dependencies):* $((C_1, j_1), \ldots, (C_n, j_n)) \models \phi^{C_i}_{j_i+1}$
  3. *for all* $i \in M$*, it holds that (state dependencies - entry):* $((C_1, j_1), \ldots, (C_n, j_n)) \models \psi^{C_i}_{j_i+1}$
  4. *for all* $i \in \{1 \ldots n\}$*, it holds that (state dependencies - invariant):* $((C_1, j'_1), \ldots, (C_n, j'_n)) \models \psi^{C_i}_{j'_i}$

*then it holds that* $Reach((C_1, j'_1, t'_1), \ldots, (C_n, j'_n, t'_n))$*, where* $j'_i = j_i + 1$ *and* $t'_i = 0$ *if* $i \in M$ *and* $j_i < |C_i|$*, and* $j'_i = j_i$ *and* $t'_i = t_i + \delta$ *otherwise.*

We define the predicate $Comp_S$ to be true iff there are no reachable states in $S$ such that no component can proceed to its next phase.

**Definition 4 (Compatibility for local requirements)** *Given the set of local requirements* $S = \{C_1 \ldots C_n\}$*, the predicate* $Comp_S$ *is true iff:*

$$\forall j_1 \in \{1 \ldots |C_1| - 1\} \ldots \forall j_n \in \{1 \ldots |C_n| - 1\} \; \forall t_1 \ldots t_n \in \mathbb{R} \Big( Reach((C_1, j_1, t_1), \ldots, (C_n, j_n, t_n)) \Rightarrow \exists M \subseteq \{1 \ldots n\} \big( M \neq \emptyset \wedge Reach((C_1, j'_1, t'_1), \ldots, (C_n, j'_n, t'_n)) \big) \Big)$$

*where* $j'_i = j_i + 1$ *and* $t'_i = 0$ *for all* $i \in M$*, or* $j'_i = j_i$ *and* $t'_i = t_i$ *otherwise. If* $Comp_S$ *holds, we say that* $C_1 \ldots C_n$ *are compatible, or equivalently that* $S$ *is compatible.*

For example, in Fig. 1a, predicate $Reach((A, 1, 4), (B, 1, 4))$ holds, but predicate $Reach((A, 1, 4), (B, 2, 0))$ does not, because for all $\delta \in \mathbb{R}$ and for all $S \subseteq \{1 \ldots n\}$, predicate $Reach((A, 1, 4), (B, 2, 0))$ is false.

*Strict Semantics* The above definition adopts a weakly-monotonic model of time, where discrete transitions are instantaneous and, therefore, the system may be in two different states at the same instant. The definition and the reductions to model checking and SMT can be easily adapted to have a strict semantics.

*Verification and Synthesis Problems* The core problem we address is to check if a given specification $S = \{C_1, \ldots, C_n\}$ is *compatible*, *i.e.*, if $Comp_S$ holds. The *consistency checking* problem amounts to checking if there *exists* a time point in which the final phase of all the local requirements is reached, that is it amounts to checking if the following formula holds:

$$\exists t_1 \ldots \exists t_n \; Reach((C_1, |C_1|, t_1), \ldots, (C_n, |C_n|, t_n))$$

If this is the case, then we say that $S$ is *consistent*. Finally, we can formalize the *synthesis problem* as the problem of computing (a symbolic representation of) the set: $\{S' \mid Comp_{S'} \wedge S' \preceq S\}$

### 2.1 NP-hardness

In this section, we show that the simplest of the problems defined above is already NP-hard. In fact, we show a reduction from SAT to the *consistency checking* problem.

Let $\varphi(\bar{x})$ be a Boolean formula over the variables $\bar{x} = \langle x_1 \ldots x_n \rangle$; without loss of generality, we assume $\varphi(\bar{x})$ to be in negated normal form, *i.e.*, with all the negations only in front of literals. For all $1 \leq i \leq n$, we define the local requirement corresponding to variable $x_i$ as $C_i = \langle P_1^i, P_2^i \rangle$, such that $B_{P_2^i} = [0, +\infty)$ and $\phi_{P_1^i} = \psi_{P_1^i} = \phi_{P_2^i} = \psi_{P_2^i} = \top$; the idea is to encode the values $\bot$ and $\top$ of each $x_i$ with the two phases $P_1^i$ and $P_2^i$, respectively. Moreover, we define the local requirement $G$, which will be useful as a gadget for the reduction, as follows: $G = \langle P_1^G, P_2^G \rangle$, where $P_2^G = \langle [0, +\infty), \varphi[x_i \mapsto \langle C_i, P_2^i \rangle, \neg x_i \mapsto \langle C_i, P_1^i \rangle], \top \rangle$. The specification $S^\varphi$ corresponding to the Boolean formula $\varphi(\bar{x})$ is defined as $S^\varphi = \{G, C_1, \ldots, C_n\}$. It holds that $\varphi(\bar{x})$ is satisfiable if and only if $S^\varphi$ is consistent. In fact, if $S^\varphi$ is consistent, then there exists a time point in which the signal dependency of the second phase of $G$ has been satisfied, and thus $\varphi(\bar{x})$ is satisfiable. Viceversa, let's suppose that $\varphi(\bar{x})$ is satisfiable and let $M$ be an arbitrary model of it, expressed as the set of true atoms, in which we also substitute every $x_i$ in it with the pair $\langle C_i, P_2^i \rangle$. Since the local requirements $C_1 \ldots C_n$ have no dependencies and, together with $G$, have only infinite bounds, there exists a time $t$ such that predicate $Reach((G, P_1^G, t), (C_1, P_{b_1}^G, t_1), \ldots, (C_n, P_{b_n}^n, t_n))$ is true, where for all $1 \leq i \leq n$, $b_i = 2$ and $t_i = 0$ iff $x_i \in M$ and $t_i = t$ otherwise. By definition of *Reach* (see Definition 3), this implies that $Reach((G, P_2^G, t), (C_1, P_2^1, t), \ldots, (C_n, P_2^n, t))$ holds, *i.e.*, $S$ is consistent.

In Sec. 3.2, we will give an encoding of the consistency checking problem based on SMT(DL) (*i.e.*, *Satisfiability Modulo Theory of Difference Logic*). In particular, we will show that the problem can be reduced to the satisfiability of a formula in SMT(DL). Since the latter belongs to NP [15], the consistency checking problem belongs to NP as well, having that *consistency checking* is NP-complete.

## 3 Verification

### 3.1 Reduction to Model Checking

In order to formalize the two verification problems into ones of model checking networks of timed automata, we use timed automata with shared variables. To this end, besides the clock constraints $\Xi(C)$, we define $L = \{l_{\mathcal{A}}, l_{\mathcal{B}}, \dots\}$ as a set of *location variables* (one for each automaton $\mathcal{A}$ in the network), and $\Theta(L)$ as the set of all Boolean combinations of atoms of type $l_{\mathcal{A}} = v_{\mathcal{A}}$, where $\mathcal{A}$ is a timed automata, $l_{\mathcal{A}} \in L$ and $v_{\mathcal{A}}$ is a state of $\mathcal{A}$.

**Definition 5 (Timed Automata with Shared Variables)** *A* timed automaton with shared variables *(TASV, for short)* $\mathcal{A} = \langle V_{\mathcal{A}}, v^0_{\mathcal{A}}, l_{\mathcal{A}}, C_{\mathcal{A}}, inv^{cl}_{\mathcal{A}}, inv^{loc}_{\mathcal{A}}, T_{\mathcal{A}}\rangle$ *consists of:*

- *a finite set of locations* $V_{\mathcal{A}}$*;*
- *an initial location* $v^0_{\mathcal{A}} \in V_{\mathcal{A}}$*;*
- *a location variable* $l_{\mathcal{A}}$ *with range* $V_{\mathcal{A}}$*;*
- *a finite set of clocks* $C_{\mathcal{A}}$*, where a clock is a real-valued variable;*
- *a* clock invariant $inv^{cl}_{\mathcal{A}} : V_{\mathcal{A}} \to \Xi(C_{\mathcal{A}})$ *for each location;*
- *a* location invariant $inv^{loc}_{\mathcal{A}} : V_{\mathcal{A}} \to \Theta(C_{\mathcal{A}})$ *for each location;*
- *a transition relation* $T_{\mathcal{A}} \subseteq V_{\mathcal{A}} \times 2^{C_{\mathcal{A}}} \times \Xi(C_{\mathcal{A}}) \times \Theta(L) \times V_{\mathcal{A}}$.

Given a set of clocks $C$, we denote with $\nu : C \to \mathbb{R}$ a *clock valuation*, that is a function assigning a rational value to each clock; with $\mathcal{V}_C$, we denote the set of all possible clock valuations over $C$. For $t \in \mathbb{R}$, $\nu + t$ is the clock valuation which maps every clock $c \in C$ to the value $\nu(c) + t$. For $R \subseteq C$, we define $\nu[R \mapsto 0]$ to be the valuation that maps $x$ to 0 if $x \in R$, and to $\nu(x)$ otherwise. When defining the product of two TASVs, we will deal with tuples $(l_{\mathcal{A}_1}, \dots, l_{\mathcal{A}_n})$ of location variables; in this context, we usually denote with $\lambda$ any function from the set of $n$-tuples of location variables to the set $V_{\mathcal{A}_1} \times \dots \times V_{\mathcal{A}_n}$. Moreover, we write that $\lambda \models \Phi$ (where $\Phi \in \Theta(L)$) iff $\Phi[l_{\mathcal{A}_i} \mapsto v_{\mathcal{A}_i}$, for all $1 \leq i \leq n]$ is true and $\lambda((\dots, l_{\mathcal{A}_i}, \dots)) = (\dots, v_{\mathcal{A}_i}, \dots)$. We give the semantics of a TASV in terms of traces and we define their product as described below.

**Definition 6 (Trace of a TASV)** *A* trace $\tau$ *of a TASV* $\mathcal{A} = \langle V_{\mathcal{A}}, v^0_{\mathcal{A}}, l_{\mathcal{A}}, C_{\mathcal{A}}, inv^{cl}_{\mathcal{A}}, inv^{loc}_{\mathcal{A}}, T_{\mathcal{A}}\rangle$ *is a (either finite or infinite) sequence of* states *of the form:*

$$\langle v_0, \nu_0, \lambda_0\rangle \xrightarrow{\alpha_1} \langle v_1, \nu_1, \lambda_1\rangle \xrightarrow{\alpha_2} \langle v_2, \nu_2, \lambda_2\rangle \xrightarrow{\alpha_3} \dots$$

*such that* $v_i \in V_{\mathcal{A}}$*,* $\alpha_i \in \mathbb{R} \cup \{\tau\}$*,* $\nu_i \in \mathcal{V}_{C_{\mathcal{A}}}$ *and* $\lambda_i \in \mathcal{V}_L$ *for all* $i \geq 0$*, and:*

- (initiation) $v_0 = v^0_{\mathcal{A}}$, $\nu_0(x) = 0$ *for all* $x \in C_{\mathcal{A}}$, $\nu_0 \models inv^{cl}_{\mathcal{A}}(v^0_{\mathcal{A}})$, $\lambda_0(l_{\mathcal{A}}) = v_0$ *and* $\lambda_0 \models inv^{loc}_{\mathcal{A}}(v^0_{\mathcal{A}})$*;*
- (consecution)*: for all* $i \geq 0$
  - *(timed transition) if* $\alpha \in \mathbb{R}$*, then* $v_{i+1} = v_i$ *and* $\nu_{i+1} = \nu_i + \alpha$, $\nu_i + \delta \models inv^{cl}_{\mathcal{A}}(v_i)$*, for all* $0 \leq \delta \leq \alpha$*, and* $\lambda_{i+1}(l_{\mathcal{A}}) = v_i$*;*

- *(discrete transition) if* $\alpha = \tau$ *then there is a tuple* $(v_i, R_i, \Xi_i, \Phi_i, v_{i+1}) \in T_{\mathcal{A}}$ *such that:* $\nu_i \models inv^{cl}_{\mathcal{A}}(v_i) \wedge \Xi_i$*;* $\lambda_i \models \Phi_i$*;* $\nu_{i+1} = \nu_i[R_i \mapsto 0]$*;* $\nu_{i+1} \models inv^{cl}_{\mathcal{A}}(v_{i+1})$*;* $\lambda_{i+1}(l_{\mathcal{A}}) = v_{i+1}$*, and* $\lambda_{i+1} \models inv^{loc}_{\mathcal{A}}(v_{i+1})$*.*

**Definition 7 (Product of TASVs)** *Given two TASVs* $\mathcal{A}$ *and* $\mathcal{B}$*, their product is the TASV* $\mathcal{A} \otimes \mathcal{B}$ *defined as follows:*

– $V_{\mathcal{A}\otimes\mathcal{B}} = V_{\mathcal{A}} \times V_{\mathcal{B}}$ *and* $v^0_{\mathcal{A}\otimes\mathcal{B}} = (v^0_{\mathcal{A}}, v^0_{\mathcal{B}})$*;*
– $l_{\mathcal{A}\otimes\mathcal{B}} = (l_{\mathcal{A}}, l_{\mathcal{B}})$*;*
– $C_{\mathcal{A}\otimes\mathcal{B}} = C_{\mathcal{A}} \cup C_{\mathcal{B}}$*;*
– $inv^{cl}_{\mathcal{A}\otimes\mathcal{B}}(v,u) = inv^{cl}_{\mathcal{A}}(v) \wedge inv^{cl}_{\mathcal{B}}(u)$*, for all* $(v,u) \in V_{\mathcal{A}\otimes\mathcal{B}}$*;*
– $inv^{loc}_{\mathcal{A}\otimes\mathcal{B}}(v,u) = inv^{loc}_{\mathcal{A}}(v) \wedge inv^{loc}_{\mathcal{B}}(u)$*, for all* $(v,u) \in V_{\mathcal{A}\otimes\mathcal{B}}$*;*
– *the transition relation is defined as follows:*

$$\begin{aligned} T_{\mathcal{A}\otimes\mathcal{B}} = &\{((v,u), R, \Xi, \Phi, (v',u)) \mid (v, R, \Xi, \Phi, v') \in T_{\mathcal{A}}\} \cup \\ &\{((v,u), R, \Xi, \Phi, (v,u')) \mid (u, R, \Xi, \Phi, u') \in T_{\mathcal{B}}\} \end{aligned}$$

It is worth noting that each TASV corresponds to a timed automaton defined in the standard way [1], and viceversa. We define now the TASV corresponding to a local requirement.

**Definition 8 (TASV for a Local Requirement)** *Let* $C = \langle P^C_1, \ldots, P^C_n \rangle$ *be a local requirement. We define the corresponding TASV* $\mathcal{A} = \{V_{\mathcal{A}}, v^0_{\mathcal{A}}, l_{\mathcal{A}}, C_{\mathcal{A}}, inv^{cl}_{\mathcal{A}}, inv^{loc}_{\mathcal{A}}, T_{\mathcal{A}}\}$ *as follows:*

– *for each phase* $P^C_i$ *of local requirement* $C$*,* $v^i_{\mathcal{A}}$ *is the corresponding location in* $V_{\mathcal{A}}$*;* $P^C_0$ *corresponds to* $v^0_{\mathcal{A}}$ *and* $C_{\mathcal{A}} = \{c_{\mathcal{A}}\}$*;*
– *for each phase* $P^C_i$ *(but the last) of* $C$*,* $inv^{cl}_{\mathcal{A}}(v^i_{\mathcal{A}}) := c_{\mathcal{A}} \leq u_{P^C_{i+1}}$*;*
– (discrete transition) *for each phase* $P^C_i$ *(but the last) of* $C$*, it holds that* $(v^i_{\mathcal{A}}, \{c_{\mathcal{A}}\}, \Xi^C_i, \Phi_{P^C_{i+1}} \wedge \Psi_{P^C_{i+1}}, v^{i+1}_{\mathcal{A}}) \in T_{\mathcal{A}}$*, where* $\Xi^C_i := l_{P^C_{i+1}} \leq c_{\mathcal{A}} \leq u_{P^C_{i+1}}$*.*
– (state deps) *for each phase* $P^C_i$ *of* $C$*, it holds that* $inv^{loc}_{\mathcal{A}}(v^i_{\mathcal{A}}) := \Psi_{P^C_i}$*;*

*where* $\Phi_P := \phi_P[(d,j) \mapsto (l_d = v_j)]$*, for each phase* $P$ *(the same holds for* $\Psi$*);*

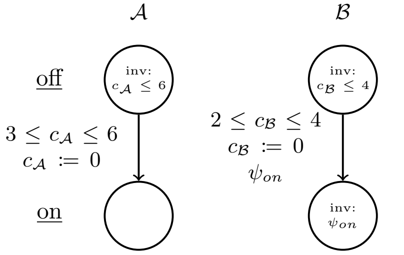

Fig. 2: Example of TASV corresponding to a local requirement.

*Example.* Consider Fig. 1a: the corresponding TASV is depicted in Fig. 2. Each phase of each local requirement corresponds to a location of the corresponding TASV; in the example, phase *off* is mapped into location off. The first locations of automata $\mathcal{A}$ and $\mathcal{B}$ have attached the invariants $c_{\mathcal{A}} \leq 6$ and $c_{\mathcal{B}} \leq 4$, respectively. Automaton $\mathcal{A}$ proceeds to location *on* (corresponding to phase $A.on$) by a transition labelled with clock constraint $3 \leq c_{\mathcal{A}} \leq 6$ and clock reset $c_{\mathcal{A}} := 0$. Since the second phase of local requirement $A$ has no dependencies, the transition to on has no constraints on the location variables. The situation is different for automaton $\mathcal{B}$, for which the transition to on is labelled with $2 \leq c_{\mathcal{B}} \leq 4$ and $c_{\mathcal{B}} := 0$, and also with $\psi_{on} := (l_{\mathcal{A}} = on)$, that is the state dependency of phase $B.on$; moreover, $\psi_{on}$ is also an invariant for the second location of automaton $\mathcal{B}$, since it is a state dependency.

Given a network $\mathcal{S} := \mathcal{A}_1 \times \cdots \times \mathcal{A}_n$ of TASVs, the problem of *consistency checking* can be expressed as the reachability of location $(\mathcal{A}_1.last, \ldots, \mathcal{A}_n.last) \in V_{\mathcal{S}}$. A *deadlock* of a TASV $\mathcal{A}$ is defined as a state $(v, t) \in V_{\mathcal{A}} \times \mathbb{R}$ such that $\mathcal{A}$ can take neither a timed nor a discrete transition from $(v, t)$. We call *livelock* a state $(v, t)$ such that $\mathcal{A}$ can take only timed transitions. The *compatibility checking* problem can be expressed as the problem of checking if there exists a trace of $\mathcal{S}$ such that (i) either the trace is finite and its final state is a deadlock of $\mathcal{S}$; we can check this property by adding a *sink* location to the TASV $\mathcal{S}$ to which all locations can transition to and by checking the reachability of it; (ii) or the trace is infinite and there exists a location $v \in V_{\mathcal{S}}$ and a point $k \geq 0$ such that $l_{\mathcal{S}} = v \neq (\mathcal{A}_1.last, \ldots, \mathcal{A}_n.last)$, for all the states after $k$ in the trace, where the $i^{th}$ component of $v$ together with the time of the current state is a *livelock* for automata $\mathcal{A}_i$, for some $1 \leq i \leq n$. The second point is fundamental for local requirements featuring *infinite bounds*: in these automata, it is not sufficient to check for deadlocks, since a timed transition could be always enabled; instead, an illegal state can be described by a trace of the system that reaches a *livelock* whose location has no invariants attached and then stays constantly in this location. Having reached a livelock, the automaton can proceed only with timed moves: in particular, it can't proceed to the next location because its dependencies are violated. We can check the second point in this way: we first add a sink location $sink_v^{\mathcal{A}_i}$ for each location $v \in \mathcal{A}_i$ (and of course a transition from the latter to the former), for each $1 \leq i \leq n$, and we attach to it the invariant $\neg inv_{\mathcal{A}_i}^{loc}(v)$. Now, in the product $S$ of these modified automata, we look for a trace such that, from a certain time point onwards, it stays constantly in a location $(l_1, \ldots, l_n)$ such that at least one $l_i$ is a sink state. This property can be formalized in *Linear Temporal Logic* as $FG(\bigvee_{1 \leq i \leq n, v \in \mathcal{A}_i} sink_v^{\mathcal{A}_i})$.

#### 3.2 Encoding into SMT(DL)

We describe the encoding into SMT(DL) (Satisfiability Modulo Theory of Difference Logic) for the problems of *consistency checking* and *compatibility checking*. For all $1 \leq c \leq n$ and $1 \leq i \leq |c|$, we introduce the following variables: (i) $r_i^c \in \mathbb{B}$ represents the fact that phase $i$ of local requirement $c$ is *reachable*; (ii) $s_i^c = (t_i^c, p_i^c)$ represents the superdense time instant in which local requirement $c$ enters phase $i$, where $t_i^c \in \mathbb{R}$ and $p_i^c \in \mathbb{N}$. We can compare two superdense-valued variables $(t, p)$ and $(t', p')$ with the lexicographical order, which we define as follows: $(t, p) \preceq (t', p')$ iff $t \leq t' \wedge (t = t' \rightarrow p \leq p')$. We now give the set of (conjunctively related) constraints which form our SMT(DL) encoding.

*Initialization.* Each local requirement starts in its first phase at the same time, *i.e.*, the real time point 0. Hence, for all $1 \leq c \leq n$, we add the constraint $t_0^c = 0$.

*Reachability.* For all local requirements $c$ and all phases $i$, it holds that if $i-1$ is not reachable then so is phase $i$, *i.e.*, $\neg r^c_{i-1} \rightarrow \neg r^c_i$. Moreover, we require the monotonicity over time, *i.e.*, $r^c_i \rightarrow (s^c_{i-1} \prec s^c_i)$.

*Bounds.* For all local requirements $c$ and all phases $i$, $c$ can move to $i$ only if it respects the bounds $[l^c_i, u^c_i]$ of phase $i$, namely $r^c_i \rightarrow (l^c_i \leq t^c_i - t^c_{i-1} \leq u^c_i)$. If $u^c_i = \infty$, then we add only the left-most inequality.

*Signal and State dependencies.* Consider a local requirement $c$ and one of its phases $i$. Since we have only a *finite* number of phases, we can preprocess both signal and state dependencies to remove from them all negations, as explained in the extended version of the paper [4]; this means that every atom in $\phi^c_i$ and $\psi^c_i$ occurs positive.

We want $c$ to reach $i$ only if all its signal and state dependencies are satisfied. For signal dependencies, we require the time point in which $c$ enters $i$ to be strictly greater[5]than the time point of the entry of the target phase and smaller than or equal to the time point of the exit of the target phase.

$$r^c_i \rightarrow \phi^c_i[(d,j) \mapsto (r^d_j \wedge s^d_j \prec s^c_i \preceq s^d_{j+1})]$$

Moreover, we have to guarantee that the state dependencies hold as well. In particular, if phase $i$ is reachable, then surely the time point in which $c$ enters $i$ has to be strictly greater than the time point in which the other local requirement reaches the target phase.

$$r^c_i \rightarrow \psi^c_i[(d,j) \mapsto (r^d_j \wedge s^d_j \prec s^c_i)]$$

Since state dependencies are invariant properties, *i.e.*, they have to hold for each time instant a local requirement is in a particular phase, if one state dependency is violated at some time point of phase $i-1$, then phase $i$ is not reachable. The contrapositive means that if phase $i$ is reachable, then the state dependencies of phase $i-1$ have to be invariant for phase $i-1$, namely:

$$r^c_i \rightarrow \forall \widetilde{s}(s^c_{i-1} \preceq \widetilde{s} \preceq s^c_i \rightarrow \psi^c_{i-1}[(d,j) \mapsto (r^d_j \wedge s^d_j \prec \widetilde{s} \preceq s^d_{j+1})]) \tag{1}$$

*Illegal States.* If phase $i$ of local requirement $c$ is not reachable, *i.e.*, $i$ is an *illegal state*, then there exists a time point $s^c_{ill}$ such that, for all the next (remaining) time points $\bar{s}$ between $s^c_{ill}$ and the upperbound of the transition, at least one dependency is not satisfied.

$$(r^c_{i-1} \wedge \neg r^c_i) \rightarrow \exists s^c_{ill} \forall \bar{s}(s^c_{ill} \preceq \bar{s} \preceq s^c_{i-1} + u^c_{i-1} \rightarrow \text{VIOLATION}(\bar{s})) \tag{2}$$

4 http://users.dimi.uniud.it/~luca.geatti/tricker.html

5 This allows us to model the observability of the events: $c$ first observes $d$ entering its phase $j$ and *then* moves.

where

$$\text{VIOLATION}(\bar{s}) := \neg\phi_i^c[(d,j) \mapsto (r_j^d \wedge s_j^d \prec \bar{s} \preceq s_{j+1}^d)] \vee \quad (3)$$

$$\neg\psi_i^c[(d,j) \mapsto (r_j^d \wedge s_j^d \prec \bar{s})] \vee \quad (4)$$

$$\exists\widetilde{s}(s_{i-1}^c \preceq \widetilde{s} \preceq \bar{s} \wedge \neg\psi_{i-1}^c[(d,j) \mapsto (r_j^d \wedge s_j^d \prec \widetilde{s} \preceq s_{j+1}^d)]) \quad (5)$$

We interpret $\bar{s} \preceq s_{i-1}^c + u_i^c$ as $\forall\bar{p}(\bar{s} \preceq s_{i-1}^c + (u_i^c, \bar{p}))$ and the $+$ symbol as the pairwise sum. In the case the upperbound of the transition is infinite, we simply do not add the $\bar{s} \preceq s_{i-1}^c + u_i^c$ inequality. We refer to the conjunction of all these constraints as W.

For *consistency checking*, we define $\text{END} := \bigwedge_{1 \leq c \leq n} r_{|c|}$ and we call $\text{W}^{\text{cons}}$ the conjunction of W with END. We check consistency by checking the satisfiability of $\text{W}^{\text{cons}}$.

For *compatibility checking*, we define $\text{ILL} := \bigvee_{\substack{1 \leq c \leq n \\ 1 \leq i \leq |c|}} \neg r_i^c$ and we call $\text{W}^{\text{ill}}$ the conjunction of W with ILL. We check the existence of an illegal state in the system by checking the satisfiability of $\text{W}^{\text{ill}}$, *i.e.*, $\text{W}^{\text{ill}}$ is satisfiable iff the local requirements are *not* compatible.

*Strict Semantics* In the strict semantics setting, we forbid two events to occur at the same real-time point. For strict semantics, the encoding is equal to W except that we interpret $\prec$ and $\preceq$ as $<$ and $\leq$, respectively, and all the $s_i^c$ variables as single real-valued variables $t_i^c \in \mathbb{R}$. We call S this encoding and we define $\text{S}^{\text{cons}}$ and $\text{S}^{\text{ill}}$ as above.

*Finite bounds and convex dependencies.* Despite being very close to the problem formalization, the W encoding features a high number of quantifications, also in alternation; therefore, in the general case, it is very burdensome for an SMT solver to first perform quantifier elimination on W and then to solve the resulting formula. Nevertheless, if we make some restrictions on the type of local requirements we consider, we are able to remove *upfront* all the quantifiers from W, without the need to use quantifier elimination techniques. In fact, suppose we consider only local requirements with *finite bounds* and *convex* state dependencies (see Sec. 2). We call $\widehat{\text{W}_{\text{fin}}^{\text{ill}}}$ the encoding equal to W except that Eq. (1) is replaced by:

$$r_i^c \rightarrow \psi_{i-1}^c[(d,j) \mapsto (r_j^d \wedge s_i^c \preceq s_{j+1}^d)] \quad (6)$$

and we add the following constraint:

$$(r_{i-1}^c \wedge \neg r_i^c) \rightarrow (t_i^c = t_{i-1}^c + u_{i-1}^c) \quad (7)$$

and we replace Eq. (2) with:

$$(r_{i-1}^c \wedge \neg r_i^c) \rightarrow \text{WEAKVIOL}(t_i^c) \quad (8)$$

where:

$$\begin{aligned} \text{WEAKVIOL}(t_i^c) \coloneqq\ & \neg\phi_i^c[(d,j) \mapsto (r_j^d \wedge t_j^d \leq t_i^c < t_{j+1}^d)] \vee \\ & \neg\psi_i^c[(d,j) \mapsto (r_j^d \wedge t_j^d \leq t_i^c)] \vee \\ & \neg\psi_{i-1}^c[(d,j) \mapsto (r_j^d \wedge t_i^c \leq t_{j+1}^d)]) \end{aligned} \tag{9}$$

We can prove that $\text{W}^{\text{ill}}$ and $\widehat{\text{W}^{\text{ill}}_{\text{fin}}}$ are equisatisfiable for every set of local requirements with only finite bounds and convex dependencies. Notably, there are no quantifiers in $\text{W}^{\text{ill}}_{\text{fin}}$: as said before, this makes the encoding dramatically more efficient with respect to W: in Sec. 5, we will consider only local requirements of this type. The details of the proofs are reported in the extended version of the paper in which, given that the proofs are a bit involved, we proceed incrementally, showing first how we can remove upfront the quantifiers in case of finite bounds with strict semantics, then in the case with weak semantics and finally in case of convex dependencies.

### 4 Synthesis

In this section, we tackle the *synthesis* problem, *i.e.*, computing the set of *all stronger* local requirements (as defined in Def. 2) of the initial local requirements such that their composition is *compatible*. We solve this problem by reducing it to a *parameter synthesis* problem (see [9] for a more detailed description); given a local requirement $C$, its corresponding *parametric local requirement* $\langle C, \pi\rangle$ is defined as $C$ (see Sec. 2), except that the bounds $l_P$ and $u_P$ of each phase $P$ are now the parameters $\bar{l}_P$ and $\bar{u}_P$, respectively, and $\pi \coloneqq \{\bar{l}_P \mid P \text{ is a phase of } C\} \cup \{\bar{u}_P \mid P \text{ is a phase of } C\}$. Given a set of local requirements $S = \{C_1, \ldots, C_n\}$, we write $\langle S, \Pi\rangle$ for its parametric version $\{\langle C_1, \pi_1\rangle, \ldots, \langle C_n, \pi_n\rangle\}$, where the set of parameters is defined as $\Pi \coloneqq \bigcup_{i=1}^n \pi_i$. A parameter valuation $\gamma : \Pi \to \mathbb{Q}$ assigns a rational value to each parameter; moreover, for each $1 \leq i \leq n$, it also induces a (concrete) local requirement $\langle C_i, \gamma(\pi_i)\rangle$, obtained from $\langle C_i, \pi_i\rangle$ by replacing every parameter $p \in \pi_i$ with the concrete value $\gamma(p)$. In the same way, we can define the *concrete* version $\langle S, \gamma(\pi)\rangle$ of $\langle S, \pi\rangle$. $\gamma$ is said to be *feasible for* $S$ if $\langle C_i, \gamma(\pi_i)\rangle$ is a *stronger* local requirement of $C_i$, for all $1 \leq i \leq n$, and $\langle S, \gamma(\pi)\rangle$ is *compatible*. A *feasible region* is a set $\mathcal{R} \coloneqq \{\gamma \mid \gamma \text{ is feasible for } S\}$. Also in this case, we can either use parameter synthesis algorithms over timed automata [3] or reduce the problem to SMT(LRA); we focus on the latter and in particular, we will synthesize a symbolic representation of the region $\mathcal{R}$, namely an SMT formula $\varphi_{\mathcal{R}}$ with the following property: $\gamma \models \varphi_{\mathcal{R}}$ iff $\gamma \in \mathcal{R}$, for each valuation $\gamma$.

Let $\overline{\text{W}^{\text{ill}}}$ be the encoding equal to $\text{W}^{\text{ill}}$ except that each number $l_i^c$ (resp. $u_i^c$) is replaced with the variable $\bar{l}_i^c$ (resp. $\bar{u}_i^c$) and each phase is required to have finite bounds. We define the sets of variables $\mathbb{R} \coloneqq \{r_i^c \mid c \in S,\ i \text{ is a phase of } c\}$ and $\mathbb{S} \coloneqq \{s_i^c \mid c \in S,\ i \text{ is a phase of } c\}$: these are the variables we are going to remove by means of quantifier elimination. Finally, we define:

$$\text{DOMAIN} := \bigwedge_{\substack{1\le c\le n\\ 1\le i\le|c|}} (\bar{l}_i^c \ge 0 \wedge \bar{l}_i^c \le \bar{u}_i^c)$$

$$\text{REFINE} := \bigwedge_{\substack{1\le c\le n\\ 1\le i\le|c|\\ u_i^c\neq\infty}} (a_i^c \le \bar{l}_i^c \wedge \bar{u}_i^c \le b_i^c)$$

The symbolic representation of the *feasible region* $\mathcal{R}$ is given by:

$$\text{SYNTH} := \text{DOMAIN} \wedge \text{REFINE} \wedge \neg\exists\mathbb{S},\mathbb{R}\left(\overline{\text{W}^{\text{ill}}}\right) \tag{10}$$

By removing the existential quantification on $\mathbb{S}$ and $\mathbb{R}$ (this can be done by means of quantifier elimination techniques), we obtain a quantifier-free formula over the variables in $\Pi$. By construction, we have that each model $\gamma$ of SYNTH is a feasible valuation, and viceversa. Therefore SYNTH is the symbolic representation of the feasible region $\mathcal{R}$.

### 5 Experimental Evaluation

We implemented the encoding described in Sec. 3.2 in a tool called TRICker (Timing Requirements Integration Checker) [6], which uses MathSAT [12] as the backend SMT engine. We compared TRICker with Uppaal [6] and Timed-nuXmv [8], both using the automata-based encoding described in Sec. 3.1.

The test set is partitioned into three categories: (i) `bounded convex` contains only systems with finite bounds and convex state dependencies; (ii) `bounded` contains systems with only finite bounds, but with arbitrary dependencies (not necessarily convex); (iii) `general` contains systems with infinite bounds and arbitrary dependencies (this is the most general fragment). Each category in turn consists of ca. 500 randomly-generated systems, divided in 10 sub-categories, namely 2c3p, 2c15p, 5c3p, 5c20p, 10c4p, 10c30p, 50c5p, 50c30p, 100c3p and 100c10p, where $N$c$M$p is the category containing only systems with $N$ components and (approximately) $M$ phases for each component. Inside each sub-category, each benchmark is randomly generated, meaning that the exact number of phases for each component and the density of its signal and state dependencies was chosen uniformly at random. For each benchmark, we compare the time spent by the three tools on the *consistency checking* and *compatibility checking* problems. We ran the experiments on a cluster of Linux machines with a 2.27GHz Xeon CPU, with a timeout of 360 seconds for each instance.

We consider first the `bounded convex` category. Fig. 3 shows the comparison of TRICker with Timed-nuXmv and Uppaal on the two verification problems. In both cases, Timed-nuXmv runs the infinite-state variant of IC3 described in [11] after discretizing the timed automata. As for Uppaal, we verify a property in the form $EF\varphi$, where $\varphi$ is a Boolean formula. For both problems, the SMT-based approach implemented in TRICker outperforms the model checkers. While there are a number of instances for which the model checkers perform better

[6] http://users.dimi.uniud.it/~luca.geatti/tricker.html

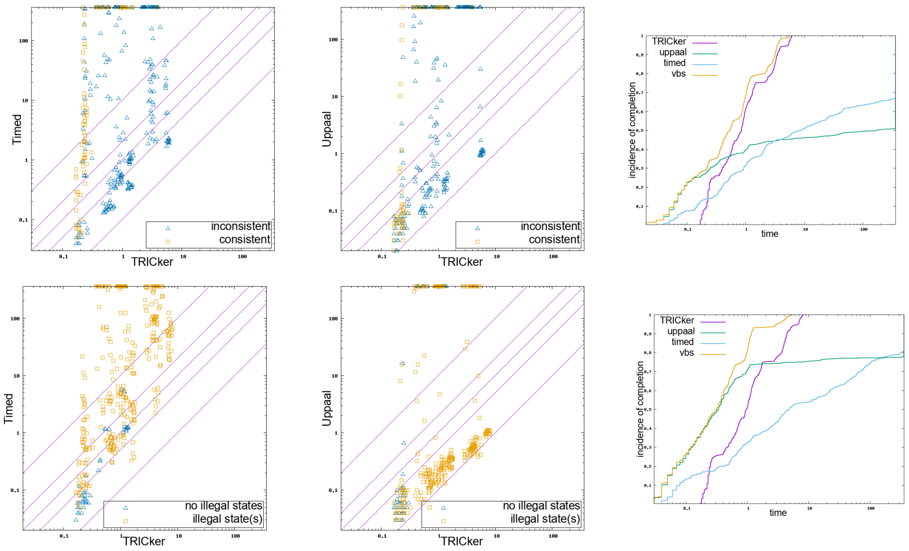

Fig. 3: Comparison on the `bounded convex` category (*consistency checking* on the first row and *compatibility checking* on the second).

than TRICker (especially for Uppaal), the latter overall solves a significantly larger amount of problems within the timeout, showing a clear improvement in scalability. This can be seen also in the survival plots comparing the three tools with the Virtual Best Solver (*vbs* for short). We can make similar considerations for the `bounded` and `general` categories, shown respectively in Fig. 4 and Fig. 5. (Note that for the `general` case, we could not evaluate Uppaal as it does not support the verification of fairness properties.) We remark that we did not note any kind of correlation between the number of signal or state dependencies in the benchmarks and the time spent by the solver. Finally, Fig. 6 shows the correlation between the memory (measured in MB) and the time (in seconds) spent by TRICker on consistency and compatibility checking, respectively.

We also evaluated the parameter synthesis algorithm described in Sec. 4. Since Uppaal currently does not support parameter synthesis for timed automata, we could not include it in the comparison. We therefore compared TRICker with Timed-nuXmv, for which we used the ParamIC3 parameter synthesis algorithm described in [9]. The algorithm is based on the inverse method, *i.e.*, it finds a bad configuration for the parameters and it tries to generalize it, maximizing the set of bad parameters removed from the current approximation of the region. We took all the consistent benchmarks of the previous test sets, which amounts to approximately 100 instances (note that for each instance of the class NcMp, the number of parameters is $\approx 2 \cdot N \cdot M$[7]). The results of the comparison are shown in Fig. 7; as in the previous cases, TRICker shows better performance and

[7] recall that both the lower and the upper bounds are parameters.

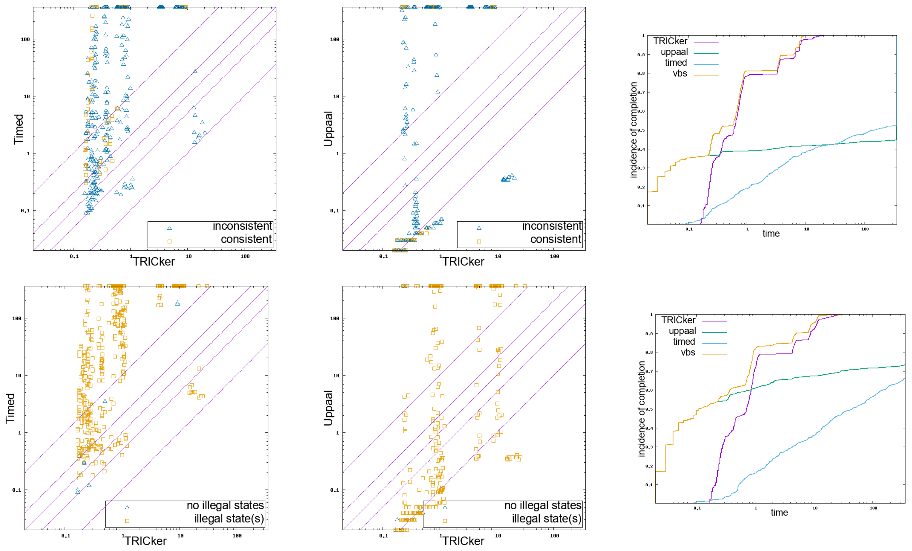

Fig. 4: Comparison on the `bounded` category (*consistency checking* on the first row and *compatibility checking* on the second).

scalability than ParamIC3, though there are several instances for which synthesis via quantifier elimination is still very expensive.

### 6 Conclusions

In this paper, we defined verification and synthesis problems of industrial relevance focused on the decomposition of startup requirements into local timing constraints and dependencies on components. Namely, we addressed the problem of checking if the local requirements are free of integration errors (i.e., consistent and compatible), and the problem of synthesizing the region of refinements of the original specification that are error free. The problem can be naturally translated into model checking and synthesis problems for timed automata with shared variables. Exploiting the structure of the requirements, we provide an encoding into SMT where consistency and incompatibility correspond to satisfiability queries, while synthesis is resolved by means of quantifier elimination.

In the future, we will consider various directions, such as extending the applicability of the approach to more general structures with loops, enriching the synthesis problem with cost functions to repair the specification driven by specific industrial goals, and considering more complex representations of signals exchanged between components.

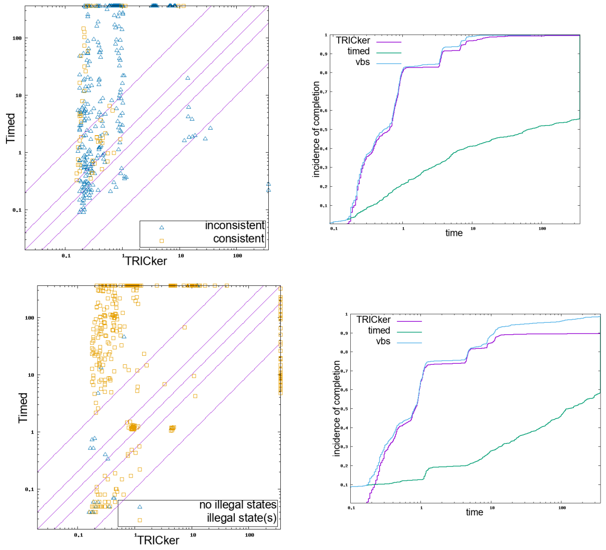

Fig. 5: Comparison on the `general` category (*consistency checking* on the first row and *compatibility checking* on the second).

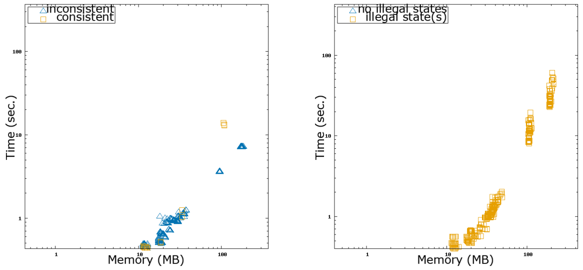

Fig. 6: Comparison between time and memory consuption of TRICker (*consistency checking* on the left and *compatibility checking* on the right).

Fig. 7: Comparison on parameter synthesis.
